# Leptin Regulates Intervertebral Disc Calcification and Ossification by Promoting Glycolysis Through OCN/HIF‐1α Axis

**DOI:** 10.1111/jcmm.71008

**Published:** 2025-12-31

**Authors:** Haoxi Li, Chengqiang Yu, Zhuhai Li, Shuyu Yao, Kaiqi Mo, Guanlu Peng, Yufeng Huang, Jianxun Wei

**Affiliations:** ^1^ Department of Spine Surgery, the People's Hospital of Guangxi Zhuang Autonomous Region, Guangxi Academy of Medical Sciences Nanning China; ^2^ Department of Spine Surgery, Shanghai East Hospital, School of Medicine Tongji University Shanghai China

**Keywords:** calcification, glycolysis, intervertebral disc degeneration, leptin, ossification

## Abstract

During intervertebral disc degeneration (IDD), cartilage endplate (CEP) cells undergo calcification and ossification, relying primarily on glycolysis for energy metabolism. Leptin (LEP) initiates the IDD, while its underlying mechanism related to glycolysis remains elusive in CEP cells. To investigate the underlying mechanism of LEP on IDD, an IDD rat model was established and LEP‐added CEP cells were adopted, with a glycolysis inhibitor (2‐DG) and sh‐HIF‐1α. A rat IDD model was successfully established, with the model group exhibiting endplate calcification, ossification, and increased LEP levels. In vitro experiments confirmed that LEP dose‐dependently promotes calcification and ossification in CEP cells. LEP also upregulated calcification‐related indicators (BMP‐2, Sox9) and osteogenesis‐related genes (OCN, Runx2), inducing the formation of calcified nodules and increased ALP activity. Mechanistic studies revealed that glycolysis‐related proteins and lactic acid content in both IDD rat and LEP‐added CEP cells were elevated. This process could be reversed using the glycolysis inhibitor 2‐DG. Further experiments demonstrated that LEP promoted glycolysis by upregulating the OCN/HIF‐1α axis; knockdown of either OCN or HIF‐1α blocked LEP‐induced calcification and ossification. LEP is elevated in IDD, and LEP accelerates the calcification and ossification through stimulating glycolysis by the OCN/HIF‐1α axis.

## 1 Introduction

Intervertebral disc (IVD) degeneration (IDD) is a leading cause of low back pain, affecting about 619 million people globally in 2020 and evaluating to over 800 million people in 2050 [1]. Current treatments largely focus on relieving pain, often fail to understand the underlying causes or prevent the IDD progression [2]. Besides impacting the individuals, IDD also causes a heavy burden on healthcare systems worldwide [3]. Consequently, it is important to investigate the underlying molecular mechanisms of IDD and exploit new therapeutic methods based on these mechanisms of IDD to improve the quality of life of patients with IDD.

IVD contained the nucleus pulposus (NP), the annulus fibrosus (AF), and the cartilage end plates (CEP) [4]. NP and AF withstand routine activities, with NP resisting axial compression while AF withstanding tensile stress; and they are supplied oxygen via diffusion through the CEP [5]. Cartilage calcification is considered to decrease articular cartilage permeability, nutrient diffusion disorder, intervertebral disc cell death, and accelerated degeneration process [6]. When calcification or ossification occurs, the increase of alkaline phosphatase (ALP), calcium, osteocalcin (OCN), and Runx2 is observed [7]. However, IDD progress is complex, and can be influenced by various factors, such as biological stress [8], aging [9], inflammation [10], and metabolic stress [11]; however, the underlying molecular mechanisms remain unelucidated.

Increasing studies indicated that accumulation of metabolites is a main reason for IDD. UHPLC–MS analysis indicated the great distinction in metabolomics between IDD and healthy IVD cells [12, 13]. Since IVD is an avascular tissue, glycolysis is the sole supply of cellular energy, which is a basic metabolic pathway that converts glucose into lactate to produce energy [14]. Recent studies demonstrated that glycolysis plays an important role in metabolic regulation and has been demonstrated to be crucial in IDD [15, 16, 17]. For example, Zhang et al. demonstrated that inhibition of glycolysis reduces NP cell senescence and inhibits IDD progression [16]. Chondrocytes also rely on an appropriate intensity of glycolysis to maintain survival and function in an avascular environment, and the glycolysis‐related regulatory axis such as Hnrnpk/Hif1α also plays important roles in the survival and differentiation of chondrocytes [18]. Therefore, we speculate that glycolysis‐related metabolic mechanisms related to might be an important regulatory mechanism for the occurrence and progression of IDD.

Leptin (LEP) is a 16‐kD non‐glycosylated peptide hormone encoded by the obese gene and plays a crucial role in the regulation of metabolic processes [19]. A study in a bovine model indicated that LEP initiates the IDD and increased the inflammatory cytokine production [20]. Han et al. also suggested that LEP can regulate CEP degeneration and ossification through the MAPK–ERK signalling pathway [21]. Inhibiting and targeting the expression of LEP can effectively inhibit osteoporosis and the IDD process [22, 23]. These findings emphasized the important involvement of LEP in IDD progression. Moreover, various studies also indicated that LEP is involved in glycolysis [24, 25, 26, 27]. However, it remains unknown whether LEP regulates the progression of the disease through glycolysis in IDD. Therefore, our study is dedicated to addressing whether and how LEP affects the calcification and ossification processes in IDD diseases by regulating glycolysis.

As for the deeper molecular mechanism by which LEP regulates glycolysis, we speculate that it might be related to the secretion of OCN and hypoxia‐inducible factor‐1α (HIF‐1α). OCN is a vitamin K‐dependent calcium binding protein synthesised and secreted by osteoblasts and serves as an important indicator of bone turnover and can reflect the status of osteoporosis [28]. Previous studies have confirmed that OCN is closely related to LEP secretion [29, 30, 31], and can regulate glucose and lipid metabolism, calcium metabolism, and cause cell calcification [32]. LEP mediates bone development and OCN secretion during the later stages of osteoblast differentiation [31]. HIF‐1α is a major regulator and mediator of glycolytic metabolism [33]. In previous findings, HIF‐1α is reported to be related to vascular calcification [34, 35]. Idelevich et al. demonstrated that OCN can promote HIF‐1α dependent glucose metabolism and cause cartilage and vascular calcification [36]. However, the correlation of OCN and HIF‐1α on calcification was mainly emphasised in the vascular system, and the roles of LEP on this IDD progression remain unknown.

Collectively, we comprehensively investigate the effects of LEP on glycolysis and intervertebral disc calcification and ossification, mainly based on presentation of OCN and HIF‐1α in IDD rat and CEP cell models. We aimed to uncover the underlying mechanisms for which LEP regulated calcification and ossification by promoting glycolysis through the OCN/HIF‐1α axis in IDD, underscoring a novel therapeutic approach for IDD.

## 2 Materials and Methods

### 2.1 Animal Modelling and Grouping

SD rats (4‐month age) were obtained from the Experimental Animal Center of Yangzhou University. The animal experiments had been reported in accordance with the ARRIVE guidelines (Animals in Research: Reporting In Vivo Experiments) [37].

After one‐week adoptive feeding, the rats underwent sham surgery (sham, *n* = 6), annular puncture (IDD model, *n* = 6), and 2‐DG (a glycolysis inhibitor) gavage (*n* = 6). All the rats were anaesthetized by a single intraperitoneal injection of ketamine at 90 mg/kg. In the control group, the anterior abdominal incision was made from both sides of the skin and peritoneum, approximately from the xiphoid process to the iliac crest, and then the anterior surface of the L3/4, L4/5, and L5/6 IVD was exposed. In the model group, injury surgery was performed using the same surgical approach as sham surgery, but exposed IVDs were punctured three times per IVD (midline front, left side, and right front) with a 26‐G puncture. Midline puncture involves injection of 2.5 μL 0.1 ng/μL TNF‐α to induce an inflammatory response. For 2‐DG treatment, 50 mg/kg of 2‐DG were intragastrically administered to rats. After 8 weeks, the rats were anaesthetized by injecting with Zoletil (0.5 g/100 g, Animal Hospital of Yangzhou University) intraperitoneally; blood was collected by cardiac puncture, plasma was separated by centrifugation, and then stored at −80°C until analysis. Finally, the rats were killed and the lumbar vertebrae were extracted for the following analysis.

### 2.2 Cell Culture, Transfection and Treatment

Rat endplate chondrocytes (EPCs) were purchased from Wuhan Pricella (CP‐R152). The cells were cultured in DMEM medium containing 1% penicillin/streptomycin and 10% FBS in conditions of 5% CO_2_ and 37°C.

After the cells were attached to the wall, 100 ng/mL LEP (HY‐P70181, MCE), 25 mM 2‐DG (HY‐13966, MCE), sh‐NC, and sh‐HIF‐1α were added separately. The EPCs were randomly generated into control, LEP, and experiment groups (2‐DG, sh‐NC, and sh‐HIF‐1α). In LEP and experiment groups, LEP (100 ng/mL) were added. In the 2‐DG group, 25 mM of 2‐DG was added. In the sh‐NC and sh‐HIF‐1α groups, EPCs were transfected with HIF‐1α shRNA (lentivirus packaging knocks down HIF‐1α) and negative control (NC) shRNA following the method in a previous study [38]. sh‐HIF‐1α was designed in the Yunzhou biological website and the sequence of sh‐HIF‐1α was CGCCTGGCCTTGGACTATATT.

### 2.3 Histological Analysis

The IVD was harvested from the sacrificed rats and stained by haematoxylin and eosin (HE) and Safranin‐O to observe the histopathology changes. Briefly, IVD were fixed with 4% paraformaldehyde for 24 h, embedded in paraffin, and then cut into 5 μm sections. For histological observation, the sections were stained with haematoxylin and 0.5% eosin solution for HE staining and stained with Safranin‐O for Safranin‐O staining.

### 2.4 Micro‐ Computed Tomography (CT) Analysis

The calcification degree of the CEP above and below the L4/5 IVD was investigated using Micro‐computed tomography (CT) (SkyScan1176, BRUKER) with a resolution of 18 μm, pressure of 65 kV, electric current of 385 μA, and exposure time of 340 ms. The 3D reconstruction and data processing were carried out using the CTVOX and CTAn software. The bone volume/tissue volume (BV/TV) was calculated for evaluation.

### 2.5 Immunohistochemical Analysis

Immunohistochemical analysis was performed to detect the Runx2 expression in NP tissue from rats. NP samples were fixed in 4% paraformaldehyde and then waxed. The wax was cut into 4‐μm‐thick sections, and the slices were cultured in citric acid buffer. Then, Runx2 primary antibodies (1:200, AF5186, Affinity) were performed to incubate the slices. 3,3′‐diaminobenzidine (DAB) and haematoxylin were served as coloration.

### 2.6 Lactic Acid Content Assay

A lactic acid assay kit (bc2230, Solarbio) was used to measure the lactic acid content. The extraction solution was added to 1 × 10^−6^ NP cells or 50 mg fresh NP tissue, and then homogenate was extracted and centrifuged to obtain the supernatant. The output was measured on a microplate reader at optical density 570 nm.

### 2.7 Alizarin Red Staining and ALP Staining

EPCs were seeded in 6‐well plates. For alizarin ref. staining, cells were fixed with 4% paraformaldehyde and stained with alizarin red S dye solution (0.2%, pH 8.3; G1450, Solarbio). For ALP staining, cells were fixed with ALP fixative and stained with Nuclear solid red staining solution.

### 2.8 Immunofluorescence

Immunofluorescence was carried out to determine the expression of Runx2 in EPC cells. Firstly, cells were put in a 12‐well plate and fixed with 4% paraformaldehyde for 10–15 min. Then, the cells were permeabilized with 1% Triton‐X 100 (T8200, Solarbio) for 5–10 min. After being blocked with 3% BSA for 30 min, the cells were stained with anti‐Runx2 antibody (1:200, AF5186, Affinity). Fluorescently labelled secondary antibody and DAPI (C1005, Beyotime) were performed for incubation for 30–60 min the next day. The immunofluorescence was visualised using a laser scanning confocal microscope.

### 2.9 Plasma Analyses

Blood was collected immediately postmortem by intracardiac puncture and plasma was separated using EDTA anticoagulation. According to the manufacturer's guide, ALP, calcium, and LEP levels were analysed using rat ALP kit (ml003360, Mlbio), Elabscience Calcium (Ca) Colorimetric test kit (ml002969, Mlbio), and rat LEP kit (E‐BC‐K103‐M, Elabscience), respectively [39, 40].

### 2.10 Quantitative Real‐Time PCR (qRT‐PCR)

qRT‐PCR was carried out to evaluate the mRNA expression of OCN and HIF‐1α. Total RNA was extracted from animal tissues and EPC cells using TRIzol reagent (15,596,018, Invitrogen). GAPDH was used as a reference. The 2^−ΔΔCt^ method was carried out to explore the relative mRNA expression levels of genes. The sequences of the primers used were listed in Table 1.

**TABLE 1:** The primers used in this study.

Name	Primer sequences (5′–3′)	Size
OCN	Forward: 5′–3′ GAGGACCCTCTCTCTGCTCA	264
	Reverse: 5′–3′ TCCTGGAAGCCAATGTGGTC	
HIF‐1α	Forward: 5′–3′ AGAACTCTCAGCCACAGTGC	219
	Reverse: 5′–3′ CTAGCAGAGTCAGGGCATCG	
GAPDH	Forward: 5′–3′ GACATGCCGCCTGGAGAAAC	92
	Reverse: 5′–3′ AGCCCAGGATGCCCTTTAGT	

### 2.11 Western Blotting

Western blotting (WB) was used to detect the protein levels (LEP, OCN, HIF‐1α, caspase3, caspase9, BMP‐2, Sox9, HK‐1, PDK1, PDHA, and p‐PDHA) both in animal tissues and EPC cells. Total protein was separated from IVD, lysed using RIPA lysis buffer (P0013B, Beyotime), and was quantified by a BCA kit (PC0020, Solarbio). GAPDH was used as an internal control. Primary antibodies used were: anti‐LEP (1:5000; L3410, Sigma‐Aldrich), anti‐OCN (1:1000; ab133612, Abcam), anti‐HIF‐1α (1:1000; ab308637, Abcam), anti‐caspase3 (1:5000; ab32351, Abcam), anti‐caspase9 (1:1000; ab32539, Abcam), anti‐BMP (1:1000; ab214821, Abcam), anti‐Sox9 (1:1000; ab185230, Abcam), anti‐HK‐1 (1:1000; #2024, CST), anti‐PDK1 (1:1000; ab202468, Abcam), anti‐PDHA (1:1000; ab168379, Abcam), and anti‐PDHA (1:1000; ab177461, Abcam).

### 2.12 Statistical Analysis

All data are expressed as mean ± standard deviation. ANOVA with Tukey's post hoc test was used for comparing the differences among multiple groups, and a t‐test was employed for comparing the differences between two groups. GraphPad 7.0 software was employed for statistical analyses and plot visualisation.

## 3 Results

### 3.1 Calcification, Ossification, and Upregulated LEP in IDD Rat

**FIGURE 1:**
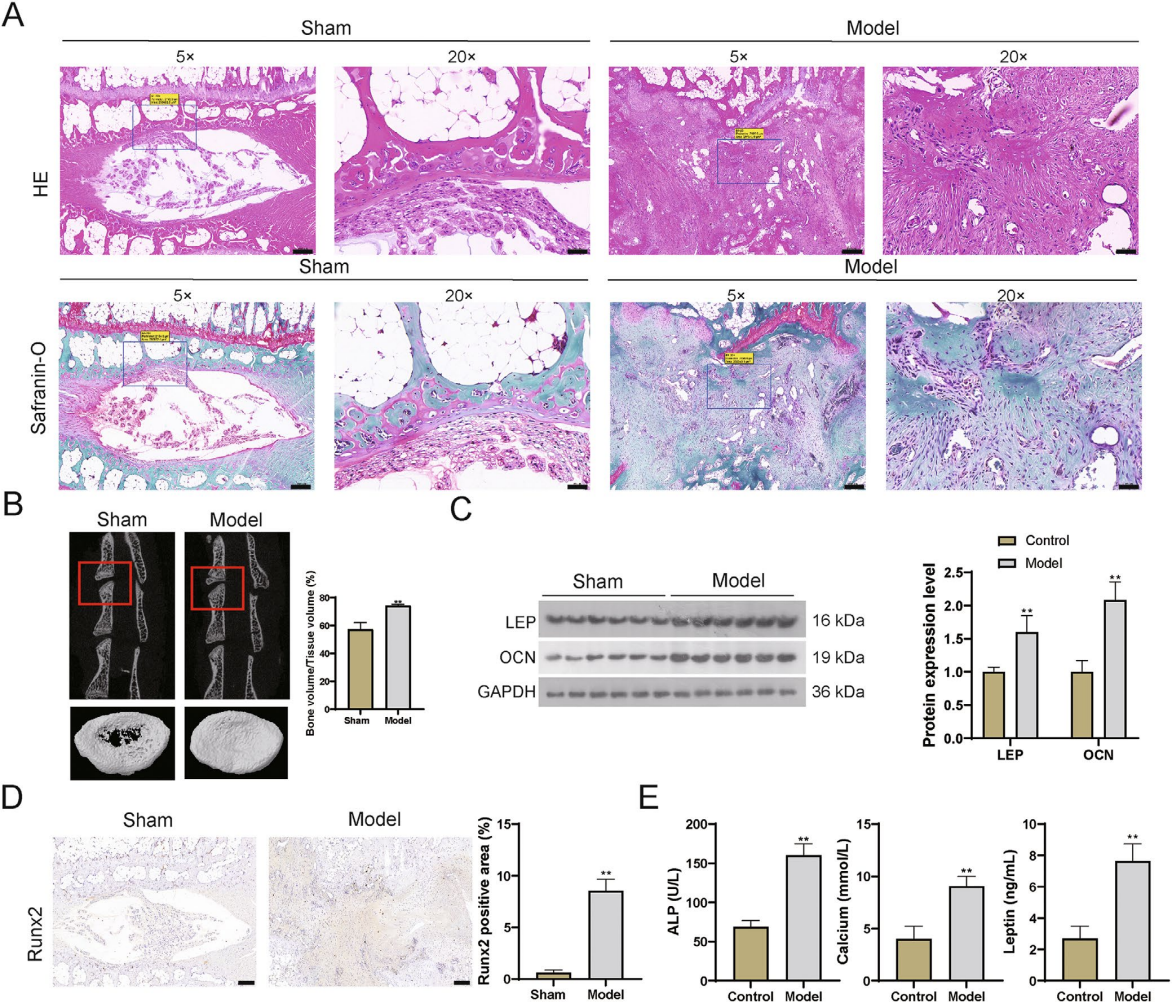
LEP is upregulated in IDD. (A) HE staining and Safranin‐O staining of L4/5 intervertebral discs. Scale bar (5×) = 200 μm; Scale bar (20×) = 50 μm. (B) Micro‐CT analysis, 3D reconstruction, and quantification of cartilage endplate (CEP) calcification via microarchitecture parameters [bone volume per tissue volume (BV/TV)]. (C) The levels of OCN and LEP detected by Western Blotting. (D) The expression levels of Runx2 detected by immunohistochemical. Scale bar = 200 μm. (E) The levels of alkaline phosphatase (ALP), calcium level, and LEP levels in blood samples. **p < 0.01 vs. control.

To figure out the potential roles of LEP in intervertebral disc calcification and ossification, an IDD model rat was established through surgical methods. HE staining and Safranin‐O staining for IVD suggested that in the sham group, the arrangement of chondrocytes in the CEP was regular; the vascular bud was abundant; and the tidemark was clear. Whereas, in the IDD model group, the CEP became thinner; chondrocytes were decreased and disorderly arranged; the tidemark advanced; the calcification and even complete ossification occurred in the CEP and epiphyseal plate near the secondary ossification center; the transition area between the CEP and the NP was replaced by many small chondrocytes; perivascular bud calcification with small lacunae was observed (Figure 1A), which demonstrated the successful construction of the IDD rat model. Moreover, CT was carried out to evaluate the calcification of the CEP above and below the L4/5 intervertebral disc. The results showed that bone volume in the model group increased when compared with the sham group (Figure 1B). In WB and immunohistochemical detections, the levels of osteogenesis‐related genes OCN and Runx2 were also upregulated in the IDD model groups using WB and immunohistochemical detections, respectively (Figure 1C,D). WB detection suggested elevated LEP in the model groups (Figure 1C). Additionally, in blood samples, ALP and calcification indicator calcium levels were also increased, followed by the increased LEP condition in the model groups (Figure 1E), which was coincident with the results in CEP tissue.

### 3.2 LEP Addition Accelerates Calcification and Ossification in Vitro

**FIGURE 2:**
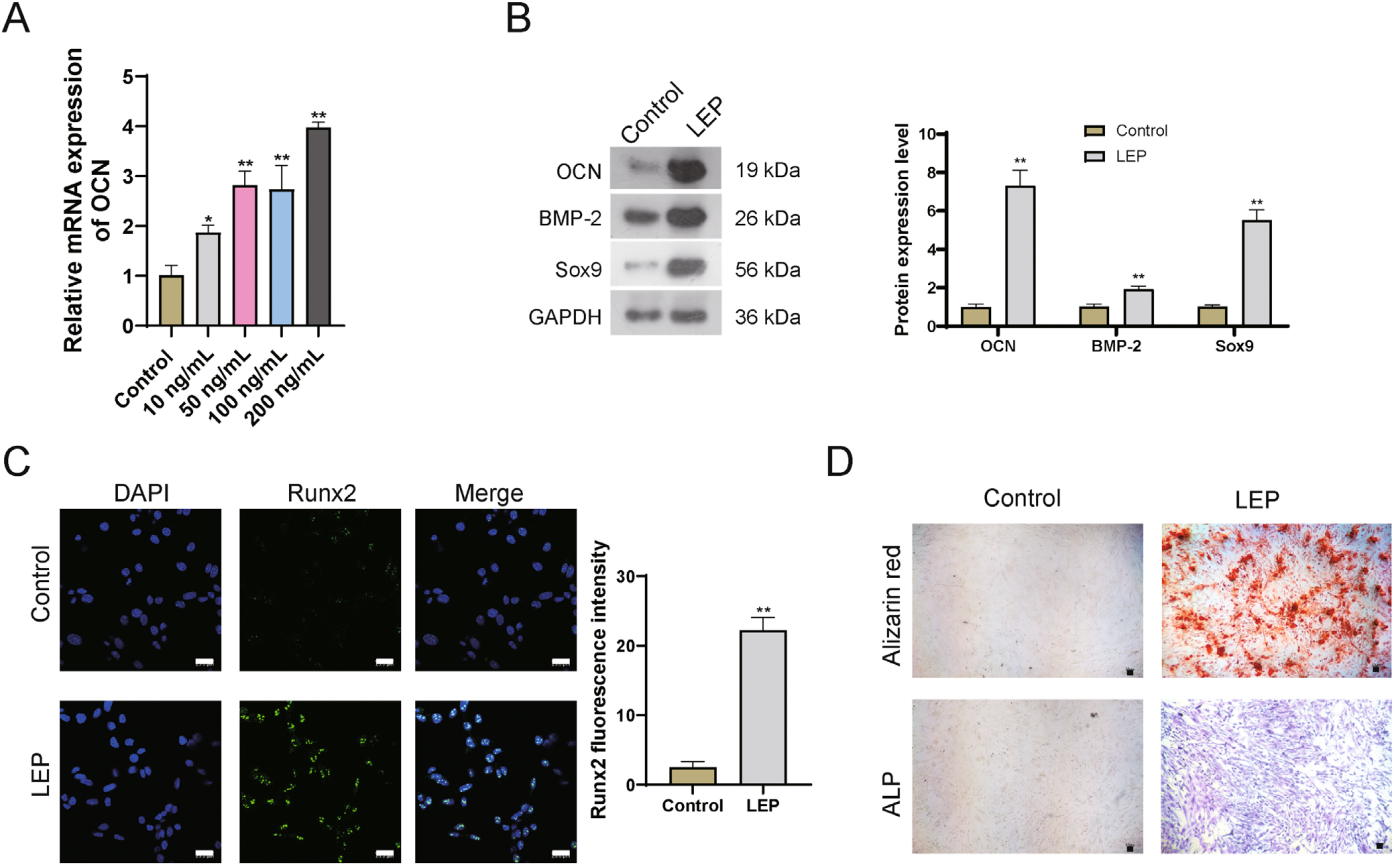
LEP addition accelerates calcification in vitro. (A) q‐PCR detection for the expression level of OCN. (B) WB detection for protein levels. (C) Immunofluorescence detection for the levels of Runx2. Scale bar = 25 μm. (D) Alizarin red staining and ALP staining for detection of calcific nodules (red) and osteocyte (blue). Scale bar = 50 μm * *p* < 0.05, ** *p* < 0.01 vs. control.

To investigate the action role of LEP in calcification and ossification, EPC cells were firstly treated with different concentrations of LEP (10, 50, 100, and 200 ng/mL). After 24‐hour treatment of LEP, the expression levels of OCN were significantly increased with a dose‐dependent manner (Figure 2A). In this study, a treatment dose of 100 ng/mL of LEP was adopted based on our findings and those of a previous study [21], as this dose was sufficient to cause a significant increase in OCN.

Furthermore, the calcification‐related index (BMP‐2 and Sox9) and osteogenesis‐related genes (OCN and Runx2) were also increased in LEP‐treated EPC cells (Figure 2B,C). Alizarin red staining and ALP staining also indicated more calcific nodules (red) and osteocyte (blue) in LEP‐treated EPCs (Figure 2D). These results implied that EPCs would undergo calcification and ossification under high‐LEP conditions.

### 3.3 LEP Accelerates Calcification and Ossification Through Glycolysis Both in IDD Rat and in Vitro EPC Cells

Considering that LEP can enhance glycolysis [27], we used 2‐DG (a glycolysis inhibitor) to intragastric administrate IDD rats and culture LEP‐treated EPC cells.

**FIGURE 3:**
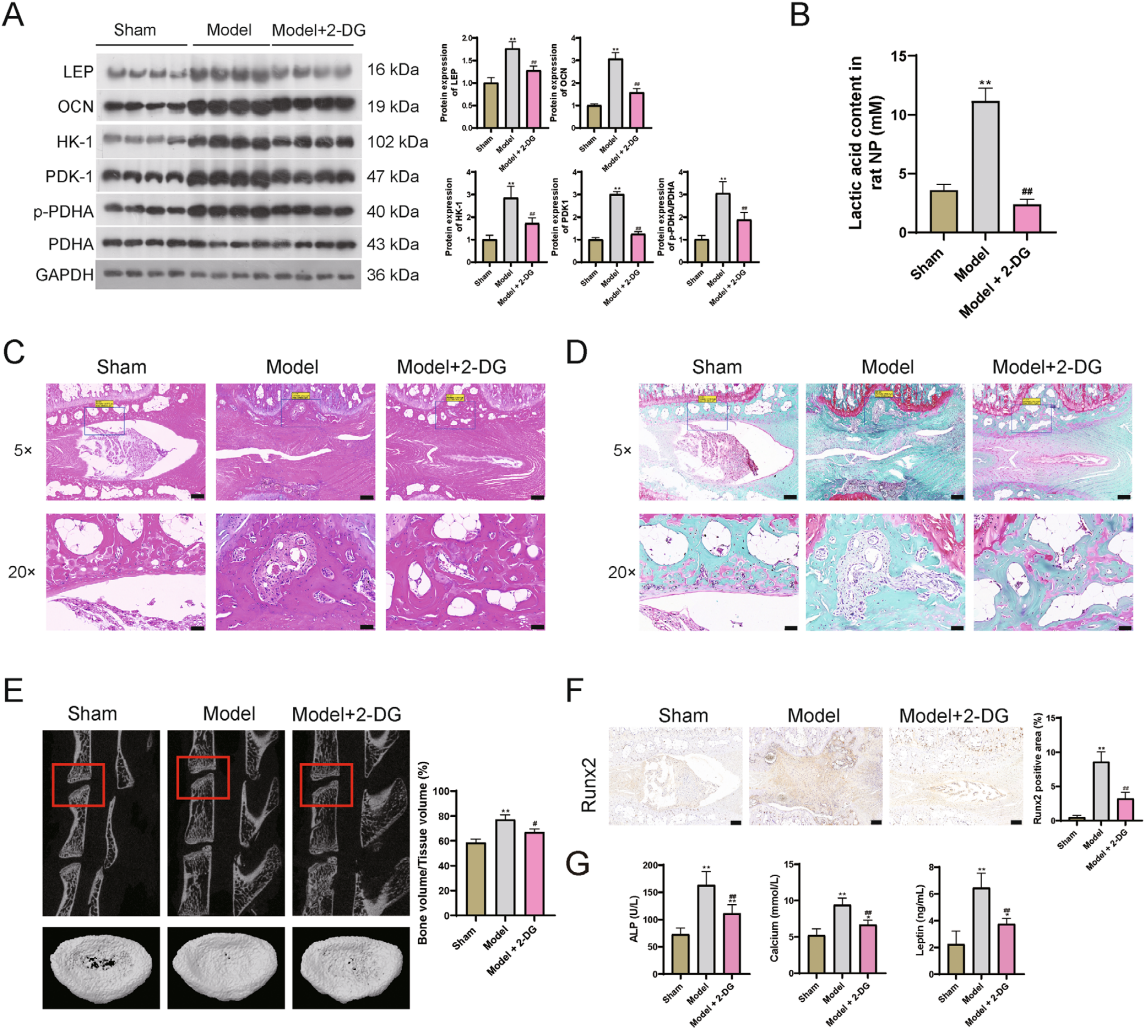
LEP accelerates calcification and ossification through glycolysis in IDD rat. (A) WB detection for protein levels. (B) Lactic acid content. (C) HE staining. Scale bar (5×) = 200 μm; Scale bar (20×) = 50 μm. (D) Safranin‐O staining. Scale bar (5×) = 200 μm; Scale bar (20×) = 50 μm. (E) Micro‐CT analysis, 3D reconstruction, and quantification of cartilage endplate (CEP) calcification via microarchitecture parameters [bone volume per tissue volume (BV/TV)]. (F) The expression levels of Runx2 detected by immunohistochemical. Scale bar = 200 μm. (G) The levels of alkaline phosphatase (ALP), calcium level, and LEP levels in blood samples. **p < 0.01 vs. sham; # *p* < 0.05 and ## *p* < 0.01 vs. model.

In the CEP tissue in rat, LEP and osteogenesis‐related genes OCN were increased in the IDD group when compared with sham; whereas down‐regulated after 2‐DG addition (Figure 3A). Glycolysis‐related proteins HK‐1, PDK1, and p‐PDHA/PDHA were also increased in the IDD group, but were also reversed by 2‐DG addition (Figure 3A). Lactic acid is the main product of anaerobic glycolysis [41], so we detect the lactic acid content in NP tissue. Elevated lactic acid was observed in IDD, while decreased lactic acid was observed in 2‐DG administration (Figure 3B). These results indicate the increase in LEP and the occurrence of glycolysis in the IDD model; however, when stimulated with 2‐DG, the expression of LEP and glycolysis were both inhibited. Additionally, as the LEP decrease, the degeneration degree of intervertebral disc was also alleviated after 2‐DG treatment in HE and Safranin‐O staining (Figure 3C,D). Moreover, CT detection showed a reduced bone volume in the 2‐DG group which was induced by IDD operation (Figure 3E). Finally, the level of osteogenesis‐related genes Runx2 were also down‐regulated under 2‐DG treatment (Figure 3F). Analogously, 2‐DG addition also decreased the ALP, calcium, and LEP levels in the model groups (Figure 3G). These results indicated that LEP accelerates calcification and ossification through glycolysis process in IDD rat.

**FIGURE 4:**
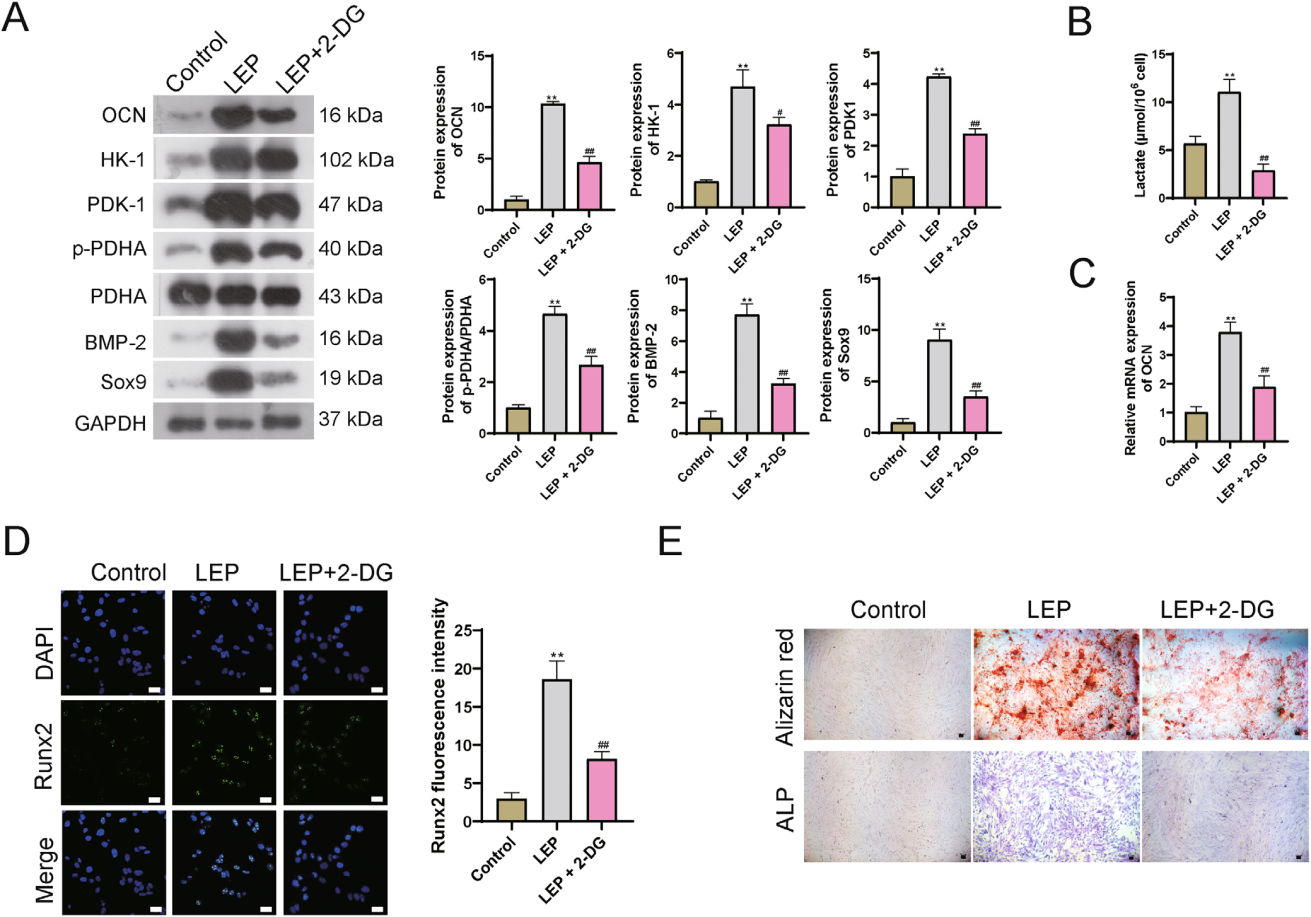
LEP accelerates calcification and ossification through glycolysis in vitro. (A) WB detection for protein levels. (B) Lactic acid content. (C) q‐PCR detection for the expression level of OCN. (D) Immunofluorescence detection for the levels of Runx2. Scale bar = 25 μm. (E) Alizarin red staining and ALP staining for detection of calcific nodules (red) and osteocyte (blue). Scale bar = 50 μm. **p < 0.01 vs. control; # *p* < 0.05 and ## *p* < 0.01 vs. model.

In the EPC cells, calcification‐related index BMP‐2 and Sox9 and glycolysis‐related proteins HK‐1, PDK1, and p‐PDHA/PDHA were also increased in LEP groups; whereas they were decreased in 2‐DG‐treated cells when compared with the LEP group (Figure 4A). Elevated lactic acid induced by LEP addition was also decreased after 2‐DG addition (Figure 4B). Osteogenesis‐related genes OCN and Runx2 were also decreased in 2‐DG‐treated EPC cells (Figure 4A,C,D). Alizarin red staining and ALP staining also indicated mitigation effects of calcific nodules (red) and osteocyte (blue) in the 2‐DG‐treated cells (Figure 4E). These results further validated that LEP accelerates calcification and ossification through the glycolysis process in vitro.

### 3.4 LEP Regulate Glycolysis in Disc Degeneration Through OCN/HIF‐1α in Vitro

**FIGURE 5:**
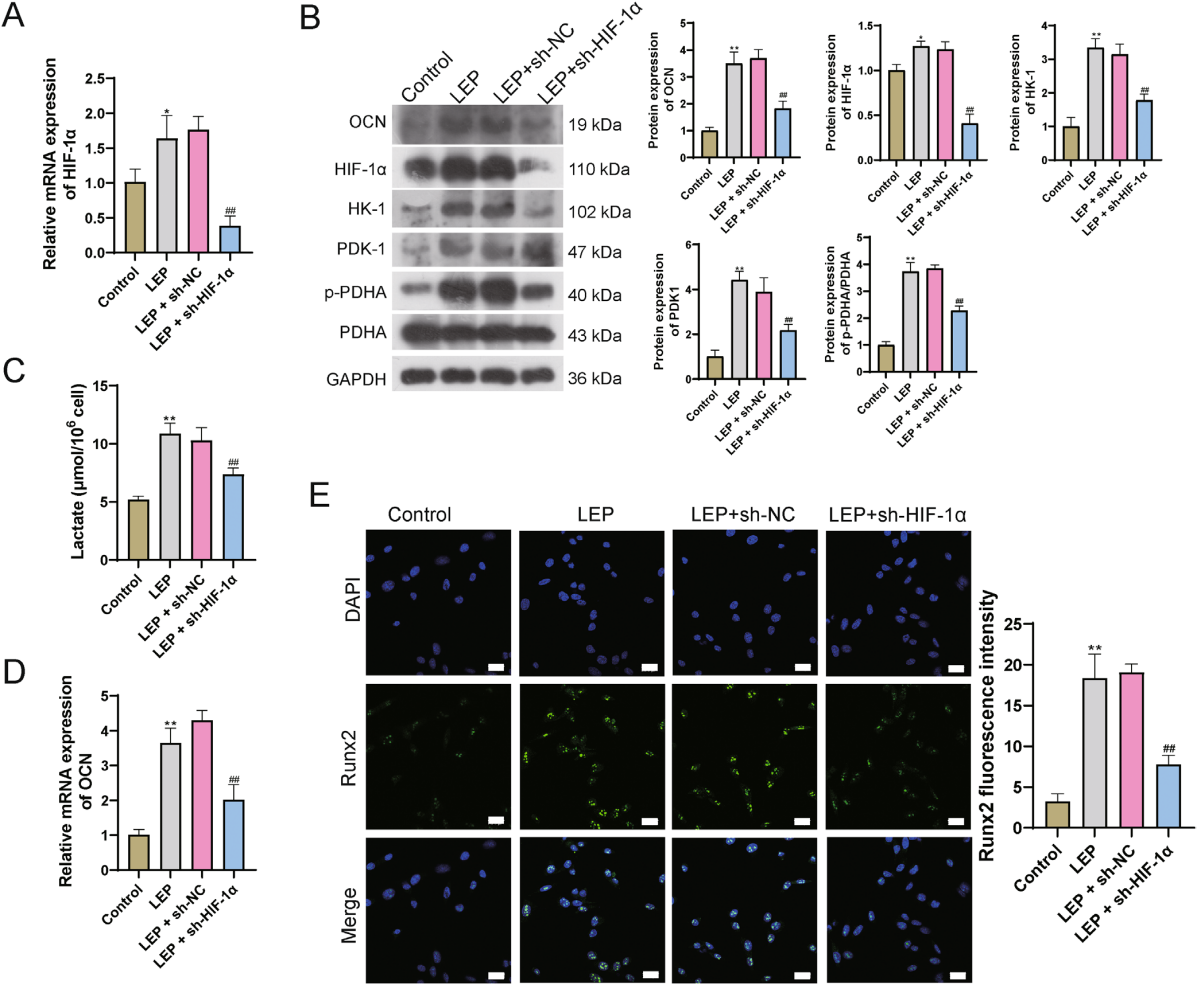
LEP regulates glycolysis in disc degeneration through OCN/HIF‐1α in vitro. (A) q‐PCR detection for the expression level of HIF‐1α. (B) WB detection for protein levels. (C) Lactic acid content. (D) q‐PCR detection for the expression level of OCN. (E) Immunofluorescence detection for the levels of Runx2. Scale bar = 25 μm. **p < 0.01 vs. control; ## *p* < 0.01 vs. LEP + sh‐NC.

Previous studies indicated that HIF‐1α is upregulated synergia with in calcification and ossification [42, 43] to regulate glycolysis [44]. Therefore, we hypothesize that LEP up‐regulates OCN, further regulates HIF‐1α to promote glycolysis, and affects articular cartilage calcification. Previous studies show that HIF‐1α is upregulated synergia with in calcification and ossification [42]. In our study, upregulated HIF‐1α was also observed in LEP‐treated cells when compared with control (Figure 5A,B). Next, we down‐regulated the HIF‐1α in LEP‐treated cells. HIF‐1α mRNA expression and protein level were significantly decreased in the sh‐HIF‐1α group (Figure 5A,B). Furthermore, glycolysis‐related proteins HK‐1, PDK1, and p‐PDHA/PDHA were decreased in the sh‐HIF‐1α group (Figure 5B). Elevated lactic acid induced by LEP addition was also decreased in the sh‐HIF‐1α group (Figure 5C). Osteogenesis‐related genes OCN and Runx2 expression were also blocked in sh‐HIF‐1α transfection groups (Figure 5B,D,E).

**FIGURE 6:**
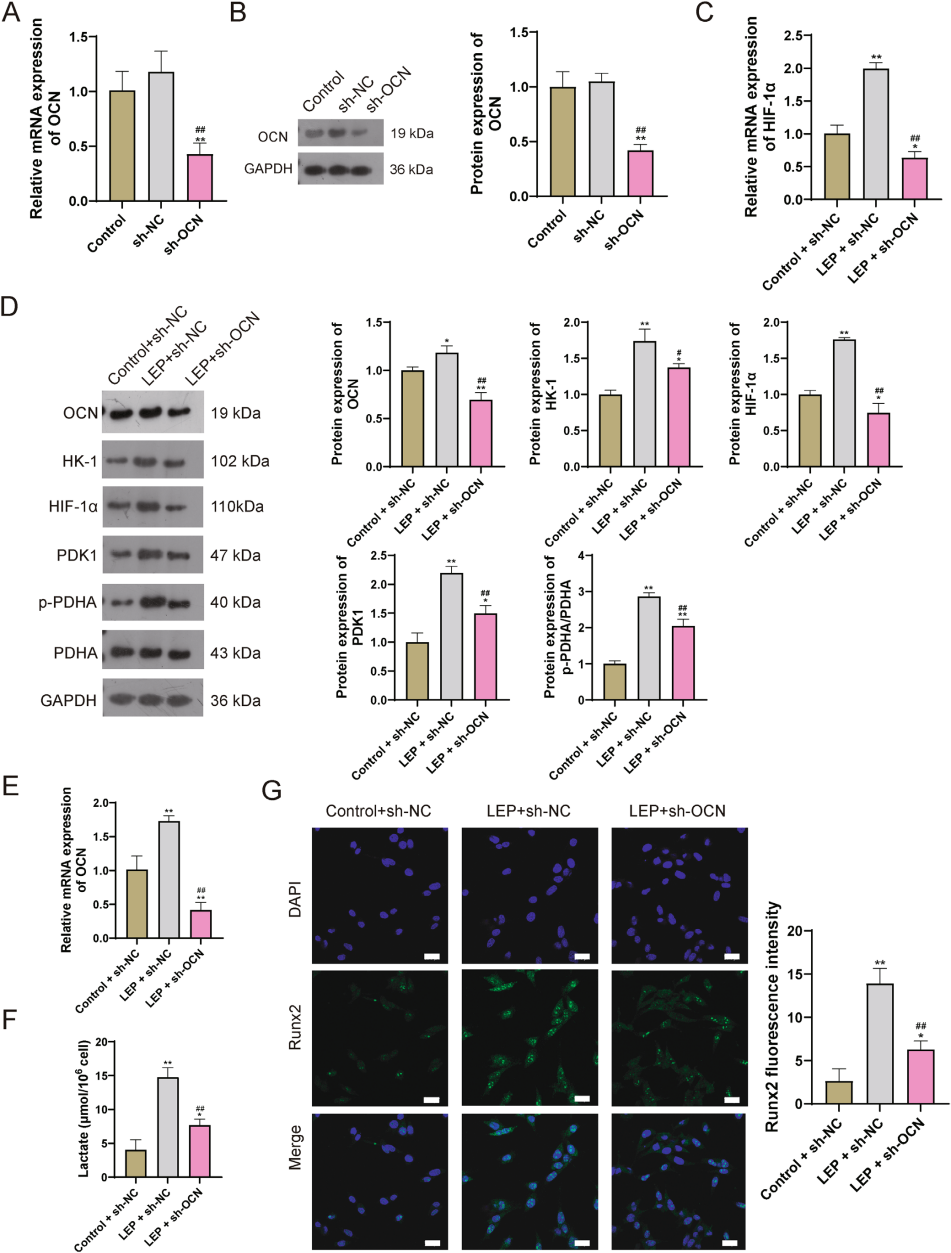
LEP regulates glycolysis in disc degeneration through OCN/HIF‐1α in vitro. (A) q‐PCR detection for the expression level of OCN. (B) WB detection for protein levels of OCN. (C) q‐PCR detection for the expression level of HIF‐1α. (D) WB detection for protein levels. (E) q‐PCR detection for the expression level of OCN. (F) Lactic acid content. (G) Immunofluorescence detection for the levels of Runx2. Scale bar = 25 μm. **p < 0.01 vs. control; ## *p* < 0.01 vs. sh‐NC/LEP + sh‐NC.

Moreover, OCN knockdown was proceeded to investigate whether LEP regulated glycolysis through OCN‐mediated HIF‐1α in vitro in disc degeneration. Significantly, OCN was successfully inhibited in EPC cells (Figure 6A,B). Moreover, the elevated downstream HIF‐1α, glycolysis‐related proteins (HK‐1, PDK1, and p‐PDHA/PDHA), lactic acid levels, osteogenesis‐related genes (OCN and Runx2) induced by LEP were reversed by OCN knockdown (Figure 6B–G). These results further validated that LEP accelerates calcification and ossification through the glycolysis process by regulating the OCN/HIF‐1α axis in vitro.

## 4 Discussion

**FIGURE 7:**
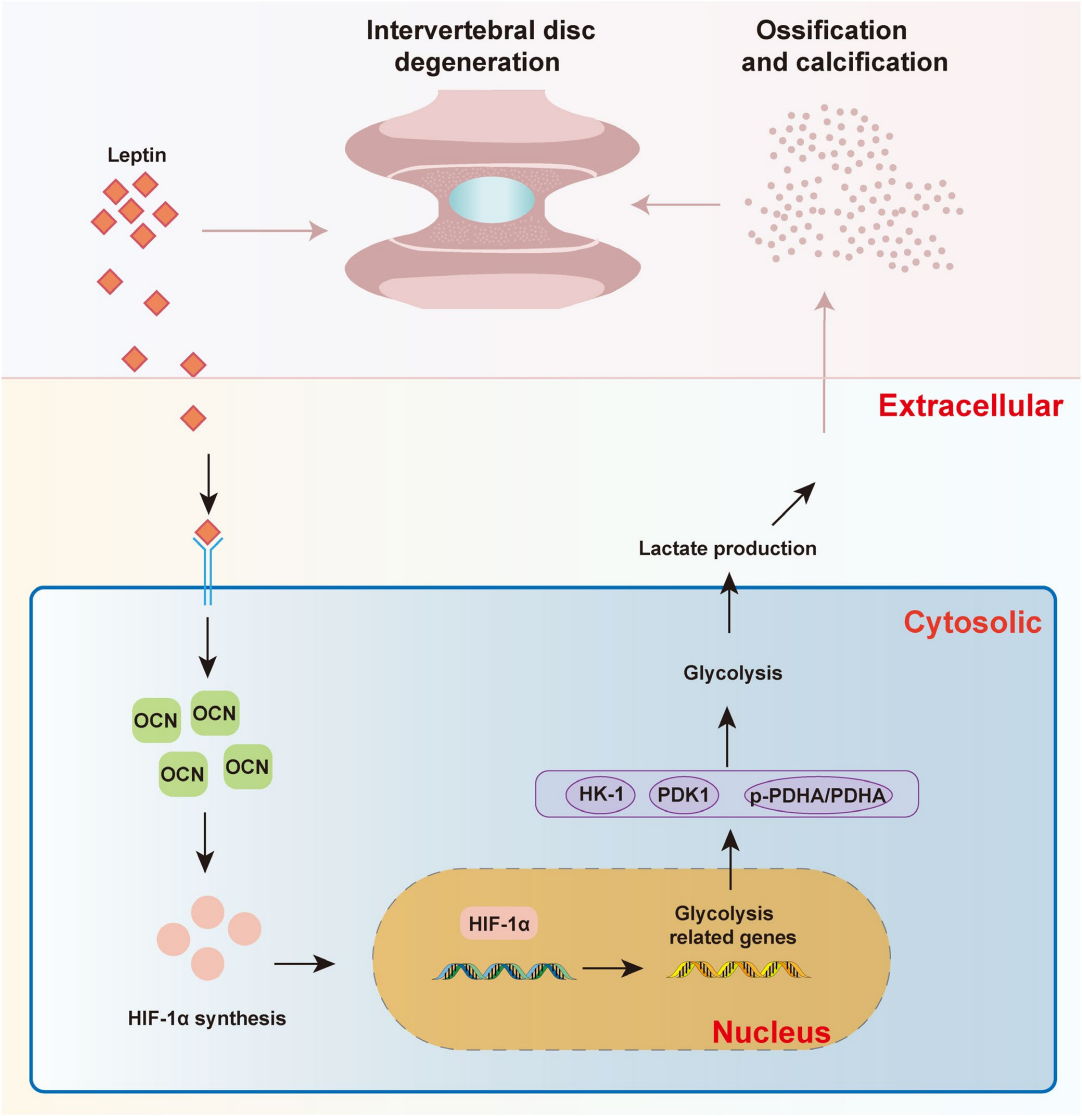
Leptin accelerates the intervertebral disc ossification and calcification by promoting mediated glycolysis through the OCN/HIF‐1α axis.

The prevalence and severity of IDD is high, but there are no effective curative treatments, largely due to a limited knowledge on its pathogenesis. In this study, we underscore the crucial role of LEP in IDD progression and demonstrated that LEP can accelerate the intervertebral disc calcification and ossification by promoting glycolysis through the OCN/HIF‐1α axis (Figure 7), based on both in vivo and in vitro experiments.

Previous studies have reported that LEP not only regulates food intake and metabolic processes, but also modulates bone formation and bone metabolism [45, 46]. In our in vivo studies, elevated LEP was observed in the IDD rat model, and increased LEP is correlated with CEP calcification and osteogenesis, showing as higher bone volume and osteogenesis‐related genes (OCN and Runx2) expression in IDD model groups. In vitro analysis also showed that LEP addition promoted the levels of osteogenesis‐related genes OCN and Runx2 and calcification‐related gene BMP‐2 and Sox9, which further indicates the degeneration of intervertebral disc and CEP cells. This result is coincident with the outcome of Han et al. [21], which also indicates that LEP is a pivotal promoter of osteoblastic differentiation in CEP cells. LEP is usually secreted from articular cartilage, skeletal growth centers, and osteoblasts, and high levels of LEP are reported to damage cartilage [47]. Increased LEP receptors in human osteoblasts and chondrocytes are observed, and LEP can bind with these receptors to directly affect bone formation and bone metabolism [48].

The underlying mechanism of LEP promoting intervertebral disc calcification and ossification might be related to the OCN/HIF‐1α axis and its‐mediated glycolysis [42, 43, 44, 49]. In our in vivo and in vitro results, LEP significantly increased the protein levels of glycolysis‐related indicators, including lactic acid level, HK‐1, PDK1, and p‐PDHA/PDHA levels. Through inhibiting the glycolysis process by adding 2‐DG, the glycolysis‐related indicators and calcification and ossification‐related indicators were all inhibited, indicating the glycolysis‐regulatory mechanism of LEP on IDD progression. The truth that leptin regulates cellular behaviour by modulating glycolysis has been proposed in various disease pathologies. For example, in mesenchymal stem cells, LEP can enhance glycolysis through OPA1‐mediated mitochondrial fusion [27]. In dendritic cells, LEP promotes cell activation through regulating the STAT3‐HK2 induced glycolysis [50]. Moreover, LEP is demonstrated to be involved in various tumour progression also through glycolysis‐related processes [26, 51, 52, 53]. Our results supplemented the acknowledgment of LEP‐regulated glycolysis in intervertebral disc calcification and ossification.

CEP is the main part of IVD and serves as crucial in nutrient supply and metabolite exchange in the IVD [54]. A study has suggested the independent links of CEP degeneration and IDD, indicating that CEP degeneration is the initial inducer contributing to IDD [55]. Zehra et al. explained that calcification of CEP influences the nutrient transport and metabolite exchange in IVD and thus promotes the IDD progression [56]. Considering the close correlation between CEP and IDD, the CEP cells were used for the underlying molecular mechanism exploration related to the OCN/HIF‐1α axis‐mediated glycolysis. In CEP cells, we demonstrated the promoting role of LEP on the OCN and other calcification and ossification indicators, which is coincident with most of the previous reports [21, 57]. At the same time as OCN increased, HIF‐1α levels in CEP also increased. And silence of HIF‐1α inhibited the degeneration of CEP cells, demonstrating that HIF‐1α is a key regulatory factor in IDD. HIF‐1α promotes bone growth mainly through two ways: (1) regulating angiogenic‐osteoblast coupling; (2) inducing metabolic reprogramming of osteoblasts, promoting anaerobic glycolysis of cells, thus providing energy supply and promoting bone repair [58]. The IVD is an avascular structure; therefore, HIF‐1α‐regulated glycolysis seems to be the most important mechanism to elevate regeneration of CEP. In our in vitro study, LEP addition increased the glycolysis process, showing as increasing lactate and high expression of glycolysis‐related proteins HK‐1, PDK1, and p‐PDHA/PDHA; while when added glycolysis inhibitor 2‐DG and sh‐HIF‐1α, the glycolysis process was inhibited. Moreover, with the inhibition of glycolysis, calcification and ossification of rat and CEP cells were also alleviated to some extent, manifesting as decreased BMP‐2, Sox9, and Runx2. In conclusion, our results further indicated that LEP can accelerate the intervertebral disc calcification and ossification by promoting glycolysis through the OCN/HIF‐1α axis.

To our knowledge, this research is the first showing the effects of LEP on the intervertebral disc and CEP cells through glycolysis, indicating that inhibition of glycolysis might be a potential approach to reduce the disc degeneration. Nevertheless, the intervertebral disc and CEP degeneration process is quite complicated; further potential mechanisms should be conducted to fully clarify our results. Moreover, how to apply this knowledge to the clinic to relieve and treat pain in patients with IDD remains a huge challenge.

## 5 Conclusion

In conclusion, this study has demonstrated that disc degeneration is correlated with elevated LEP expression, and the mechanism of LEP promoting calcification and ossification is related to the glycolysis process induced by HIF‐1α. This study uncovers the potential mechanism of LEP in IDD progression from an innovative glycolysis perspective, providing references for the further targeting drug exploitation targeted to LEP, HIF‐1α, and glycolysis in patients with IDD.

## Author Contributions

**Jianxun Wei:** conceptualization, project administration, methodology, writing – original draft, writing – review and editing.

## Funding

This study was funded by Guangxi Science and Technology Planning Project (Guike ad19245034) and Guangxi Natural Science Foundation Project Fund project (2023GXNSFAA026285).

## Ethics Statement

The animal studies have been approved by the Experimental Animal Ethics Committee of Yangzhou University on July 23, 2024 (Approval No: 202407033). All procedures involving animals were in accordance with the protocol statements and ethical principles established by the Chinese Association for Laboratory Animal Science (CALAS) Guidelines for the Care and Use of Laboratory Animals; we strictly adhere to the ethical standards governing the use and conduct of animal experiments.

## Consent

All authors of this manuscript have read and approved the final version and affirm that the work has not been published elsewhere nor is it under consideration by another journal.

## Conflicts of Interest

The authors declare no conflicts of interest.

## Data Availability

The data that support the findings of this study are available from the corresponding author upon reasonable request.
